# Effectiveness of 2024–2025 COVID-19 Vaccines in Children in the United States — VISION, August 29, 2024–September 2, 2025

**DOI:** 10.15585/mmwr.mm7440a1

**Published:** 2025-12-11

**Authors:** Stephanie A. Irving, Elizabeth A.K. Rowley, Sean Chickery, Karthik Natarajan, Nicola P. Klein, Shaun J. Grannis, Toan C. Ong, Sarah W. Ball, Malini B. DeSilva, Kristin Dascomb, Allison L. Naleway, Melissa S. Stockwell, Ashley B. Stephens, Ousseny Zerbo, John Hansen, Lawrence Block, Karen B. Jacobson, Brian E. Dixon, Colin Rogerson, Tom Duszynski, Michelle A. Barron, David Mayer, Catia Chavez, Zachary A. Weber, Sarah E. Reese, Inih Essien, Tamara Sheffield, Daniel Bride, Julie Arndorfer, Josh Van Otterloo, Padma Koppolu, Josephine Mak, Amber Kautz, Jennifer DeCuir, Ryan E. Wiegand, Amanda B. Payne, Ruth Link-Gelles

**Affiliations:** ^1^Kaiser Permanente Center for Health Research, Portland, Oregon; ^2^Westat, Inc., Bethesda, Maryland; ^3^Department of Biomedical Informatics, Columbia University Irving Medical Center, New York, New York; ^4^NewYork-Presbyterian Hospital, New York, New York; ^5^Vaccine Study Center, Division of Research, Kaiser Permanente Northern California, Oakland, California; ^6^Regenstrief Institute Center for Biomedical Informatics, Indianapolis, Indiana; ^7^Department of Family Medicine, School of Medicine, Indiana University, Indianapolis, Indiana; ^8^University of Colorado, Anschutz Medical Campus, Colorado School of Medicine, Aurora, Colorado; ^9^HealthPartners Research Institute, Minneapolis, Minnesota; ^10^Division of Infectious Diseases and Clinical Epidemiology, Intermountain Health, Salt Lake City, Utah; ^11^Division of Child and Adolescent Health, Department of Pediatrics, Columbia University, New York, New York; ^12^Department of Health Policy and Management, Richard M. Fairbanks School of Public Health, Indiana University, Indianapolis, Indiana; ^13^Department of Pediatrics, School of Medicine, Indiana University, Indianapolis, Indiana; ^14^Department of Epidemiology, Richard M. Fairbanks School of Public Health, Indiana University, Indianapolis, Indiana; ^15^National Center for Immunization and Respiratory Diseases, CDC; ^16^General Dynamics Information Technology, Atlanta, Georgia.

**Summary**

**What is already known about this topic?**

In June 2024, CDC’s Advisory Committee on Immunization Practices recommended 2024–2025 COVID-19 vaccination for all persons aged ≥6 months to provide additional protection against severe COVID-19.

**What is added by this report?**

During August 29, 2024–September 2, 2025, within a multisite network including nine states, vaccine effectiveness of 2024–2025 COVID-19 vaccination was an estimated 76% against COVID-19–associated emergency department or urgent care (ED/UC) visits among immunocompetent children aged 9 months–4 years and an estimated 56% among children and adolescents aged 5–17 years, compared with those who did not receive a 2024–2025 vaccine.

**What are the implications for public health practice?**

In a population with some persons having preexisting levels of protection from previous vaccination, previous infection, or both, 2024–2025 COVID-19 vaccination provided children with additional protection against COVID-19–associated ED/UC encounters compared with no 2024–2025 vaccination.

## Abstract

During September 2023–August 2024, approximately 38,000 COVID-19–associated hospitalizations occurred among children and adolescents aged <18 years in the United States, a rate of approximately 53 per 100,000 children, ranging from 600 per 100,000 children aged <6 months to 21 per 100,000 children and adolescents aged 5–17 years. On June 27, 2024, the Advisory Committee on Immunization Practices recommended that all persons aged ≥6 months receive a 2024–2025 COVID-19 vaccine, which targeted Omicron JN.1 and JN.1-derived sublineages. Investigators used a test-negative case-control design to estimate vaccine effectiveness (VE) of 2024–2025 COVID-19 vaccines against COVID-19–associated emergency department or urgent care (ED/UC) visits during August 29, 2024–September 2, 2025, among immunocompetent children aged 9 months–4 years and children and adolescents aged 5–17 years in the CDC-funded Virtual SARS-CoV-2, Influenza, and Other respiratory viruses Network (VISION), a multisite electronic health record–based network in nine states. Among children aged 9 months–4 years, VE against COVID-19–associated ED/UC visits was estimated at 76% (95% CI = 58%–87%) during the first 7–179 days after vaccination. Among children and adolescents aged 5**–**17 years, VE against COVID-19–associated ED/UC visits was an estimated 56% (95% CI = 35%–70%) during the first 7–179 days after vaccination. These findings suggest that vaccination with a 2024–2025 COVID-19 vaccine dose provided children with additional protection against COVID-19–associated ED/UC encounters compared with no 2024–2025 dose.

## Introduction

Since 2020, COVID-19 has accounted for thousands of hospitalizations and hundreds of deaths in the United States each season, including approximately 38,000 hospitalizations among children and adolescents aged <18 years during September 2023–August 2024 (*1*). During 2024, the SARS-CoV-2 Omicron JN.1 and JN.1-derived lineages predominated and were genomically divergent from the XBB lineages on which the 2023–2024 COVID-19 vaccines were based.* On June 27, 2024, the CDC’s Advisory Committee on Immunization Practices (ACIP) recommended 2024–2025 COVID-19 vaccines for persons aged ≥6 months (*2*); 2024–2025 COVID-19 vaccines became available on August 22, 2024.^†^ For previously unvaccinated immunocompetent children aged 6 months–4 years, a multidose initial series (including 3 doses of Pfizer-BioNTech vaccine or 2 doses of Moderna vaccine) was recommended; a single 2024–2025 dose was recommended for those who completed an initial series. For immunocompetent children aged ≥5 years, a single 2024–2025 dose was recommended, regardless of vaccination history^§^ (*2*). Per an announcement from the Secretary of the U.S. Department of Health and Human Services, the Child and Adolescent Immunization Schedule was updated in May 2025 to indicate shared clinical decision-making^¶^ for COVID-19 vaccination for healthy children and adolescents aged 6 months–17 years.**

This analysis estimated 2024–2025 COVID-19 vaccine effectiveness (VE) against COVID-19–associated emergency department or urgent care (ED/UC) encounters in children aged 9 months–4 years and children and adolescents aged 5–17 years during August 29, 2024–September 2, 2025. Age 9 months rather than 6 months was the youngest age included to allow time for completion of the Pfizer-BioNTech initial series in children aged 6 months.^††^

## Methods

### Data Source

The Virtual SARS-CoV-2, Influenza, and Other respiratory viruses Network (VISION) is a multisite electronic health record (EHR)–based network including ED/UCs and hospitals in nine states used to estimate VE. Methods for VE analyses in both adult and pediatric populations within VISION have been described (*3*–*6*). In VISION VE analyses, eligible encounters at participating health care systems are those among patients who have received molecular testing (e.g., real-time reverse transcription–polymerase chain reaction) or antigen testing for SARS-CoV-2 during the 10 days before or ≤72 hours after an eligible ED/UC encounter or hospital admission for COVID-19–like illness.^§§^ This analysis included encounters among eligible immunocompetent children and adolescents aged 9 months–17 years who visited a participating ED/UC during August 29, 2024–September 2, 2025. COVID-19 vaccination history is ascertained from state or jurisdictional registries, EHRs, and, in a subset of sites, medical claims data.^¶¶^

### Data Analysis

Eligible encounters from seven participating health care systems, including 256 ED/UCs, during August 29, 2024–September 2, 2025, were included. Case-patients were those with an ED/UC encounter for COVID-19–like illness and receipt of a positive SARS-CoV-2 molecular or antigen test result; control patients were those with an ED/UC encounter for COVID-19–like illness and receipt of a negative SARS-CoV-2 molecular test result.***

Children were excluded from analyses if they received a 2024–2025 COVID-19 vaccine dose <7 days before their index date^†††^ or received a 2024–2025 COVID-19 vaccine dose <2 months after receiving any previous COVID-19 vaccine dose, unless part of an initial series. COVID-19 case-patients were also excluded if they received a positive test result for influenza virus or respiratory syncytial virus at the time of their SARS-CoV-2 ED/UC encounter. To reduce bias from overlapping vaccination patterns, control patients who received a positive or indeterminant influenza test result were excluded from the primary analysis (*7*). Previous SARS-CoV-2 infections are incompletely documented in medical records; therefore, children were included regardless of previous SARS-CoV-2 infections.

Primary VE analyses were conducted by age groups 9 months–4 years and 5–17 years due to differences in the recommended COVID-19 vaccination schedule. In primary VE analyses, children aged 9 months–4 years were considered vaccinated if they completed an initial series with at least 1 2024–2025 dose as part of that series or completed an initial series and then received a 2024–2025 dose as an additional vaccine. The 9 months–4 years comparator group comprised children who had completed the initial COVID-19 vaccine series but had not received a 2024–2025 dose or had no recorded COVID-19 vaccination. Children aged 9 months–4 years with an incomplete initial series were excluded from the primary analysis to assess the ACIP-recommended schedule for this age group. A sensitivity analysis among children aged 9 months–4 years compared children who received at least 1 2024–2025 COVID-19 vaccine dose with children who did not, regardless of COVID-19 vaccination history. Among children and adolescents aged 5–17 years, primary VE analyses compared those who received a 2024–2025 COVID-19 vaccine dose with those who did not, regardless of COVID-19 vaccination history. Results were also stratified by age groups of 5–11 years and 12–17 years.

Odds ratios (ORs) and 95% CIs were estimated using multivariable logistic regression, comparing persons who received a 2024–2025 COVID-19 vaccine dose with those who did not among case-patients and control patients, as described in this report. Models were adjusted a priori for age in years, race and ethnicity, sex, calendar day (days since August 29, 2024, to account for variability in COVID-19 circulation), and geographic region with age and calendar day included as natural splines.^§§§^ VE was calculated as (1 − adjusted OR) x 100% during the first 7–179 days since receipt of the most recent 2024–2025 COVID-19 vaccine dose. Sensitivity analyses in both the 9 months–4 years and 5–17 years age groups examined VE during the 7–299 days since receipt of a 2024–2025 COVID-19 vaccine dose.

Analyses were conducted using R software (version 4.3.2; R Foundation). This activity was reviewed by CDC, deemed not research, and was conducted consistent with applicable federal law and CDC policy.^¶¶¶^

## Results

### 2024–2025 COVID-19 VE Against COVID-19–Associated ED/UC Visits in Children Aged 9 Months–4 Years

Among children aged 9 months–4 years, 44,541 ED/UC encounters met criteria for inclusion in the analyses, including 1,292 (3%) case-patients and 43,249 (97%) control patients (Table 1). Twelve (<1%) case-patients and 1,847 (4%) control patients had received a 2024–2025 COVID-19 vaccine dose. Effectiveness of a 2024–2025 COVID-19 vaccination against a COVID-19–associated ED/UC visit was 76% (95% CI = 58%–87%) during the first 7–179 days after vaccination and 77% (95% CI = 62%–86%) during the first 7–299 days after vaccination (Table 2). VE point estimates were lower at 66% when the comparator group was expanded to include children with an incomplete initial COVID-19 vaccination series, but CIs overlapped with those in the primary analysis (95% CI = 51%–76%).

**TABLE 1:** Patient characteristics of children and adolescents aged 9 months**–**17 years with COVID-19–associated emergency department or urgent care encounters, by age group — VISION, August 29, 2024–September 2, 2025

Characteristic	No. (column %)		
	Total encounters	COVID-19 case-patients	COVID-19 control patients
**Age 9 mos–4 yrs**	**44,541 (100)**	1,292 (100)	43,249 (100)
HHS region*			
2	**5,208 (12)**	113 (9)	5,095 (12)
5	**15,589 (35)**	469 (36)	15,120 (35)
8	**6,613 (15)**	194 (15)	6,419 (15)
9	**15,575 (35)**	461 (36)	15,114 (35)
10	**1,556 (3)**	55 (4)	1,501 (3)
Female sex	**19,648 (44)**	564 (44)	19,084 (44)
Race and ethnicity			
Black or African American, non-Hispanic	**6,008 (13)**	139 (11)	5,869 (14)
White, non-Hispanic	**15,855 (36)**	476 (37)	15,379 (36)
Hispanic or Latino, any race	**14,199 (32)**	391 (30)	13,808 (32)
Other, non-Hispanic^†^	**7,390 (17)**	247 (19)	7,143 (17)
Unknown**^§^**	**1,089 (2)**	39 (3)	1,050 (2)
2024–2025 COVID-19 vaccine receipt^¶^	**1,859 (4)**	12 (1)	1,847 (4)
Moderna	**396 (21)**	2 (17)	394 (21)
Pfizer-BioNTech	**1,463 (79)**	10 (83)	1,453 (79)
**Age 5–17 yrs**	**53,467 (100)**	1,325 (100)	52,142 (100)
HHS region*			
2	**4,857 (9)**	77 (6)	4,780 (9)
5	**18,833 (35)**	484 (37)	18,349 (35)
8	**12,994 (24)**	424 (32)	12,570 (24)
9	**14,609 (27)**	291 (22)	14,318 (27)
10	**2,174 (4)**	49 (4)	2,125 (4)
Female sex	**26,561 (50)**	687 (52)	25,874 (50)
Race and ethnicity			
Black or African American, non-Hispanic	**7,290 (14)**	166 (13)	7,124 (14)
White, non-Hispanic	**22,896 (43)**	603 (46)	22,293 (43)
Hispanic or Latino, any race	**14,961 (28)**	346 (26)	14,615 (28)
Other, non-Hispanic^†^	**7,585 (14)**	193 (15)	7,392 (14)
Unknown**^§^**	**735 (1)**	17 (1)	718 (1)
2024–2025 COVID-19 vaccine receipt^¶^	**2,488 (5)**	26 (2)	2,462 (5)
Moderna	**434 (17)**	5 (19)	429 (17)
Novavax	**5 (0)**	0 (—)	5 (0)
Pfizer-BioNTech	**2,049 (82)**	21 (81)	2,028 (82)

**Abbreviations:** HHS = U.S. Department of Health and Human Services; VISION = Virtual SARS-CoV-2, Influenza, and Other respiratory viruses Network.

* In VISION, geographic region was included in the model based on site of the final discharge facility of the encounter. HHS regions are included to illustrate geographic spread across VISION; regions are defined by HHS. States included in each region are available at HHS Regional Offices | HHS.gov. Included VISION sites were: *Region 2:* Columbia University (New York); *Region 5:* HealthPartners (Minnesota and Wisconsin) and Regenstrief Institute (Indiana); *Region 8*: Intermountain Healthcare (Utah) and University of Colorado (Colorado); *Region 9*: Kaiser Permanente Northern California (California); and *Region 10*: Kaiser Permanente Northwest (Oregon and Washington).

^†^ “Other, non-Hispanic” race includes persons reporting non-Hispanic ethnicity and any of the following for race: American Indian or Alaska Native, Asian, Middle Eastern or North African, Native Hawaiian or Pacific Islander, other races not listed, and multiple races. Because of small numbers, these categories were combined.

**^§^** “Unknown” includes persons with missing race and ethnicity in their electronic health record.

^¶^ Receipt of a 2024–2025 COVID-19 vaccine dose during the 7–179 days before the index date (defined as the emergency department or urgent care encounter or respective SARS-CoV-2 testing date, whichever came first).

**TABLE 2:** Vaccine effectiveness* against laboratory-confirmed COVID-19–associated emergency department or urgent care encounters among children aged 9 months–4 years — VISION, August 29, 2024–September 2, 2025

COVID-19 vaccination pattern	Total encounters, no.	Days since last dose among vaccinated persons, no.		Positive SARS-CoV-2 test results, no. (column %)	VE, % (95% CI)	
		Median (IQR)	Maximum		Unadjusted	Adjusted*
**Primary VE estimates^†^**						
No 2024–2025 COVID-19 dose (Ref)	**42,682**	407 (290–675)	1,474	1,280 (99)	Ref	Ref
≥ 1 2024–2025 COVID-19 dose, 7–179 days earlier	**1,859**	78 (42–118)	179	12 (1)	79 (63–88)	76 (58–87)
**Sensitivity VE estimates**						
Extended VE interval^†,§^						
No 2024–2025 COVID-19 dose (Ref)	**42,682**	407 (290–675)	1,474	1,280 (99)	Ref	Ref
≥ 1 2024–2025 COVID-19 dose, 7–299 days earlier	**2,207**	91 (49–147)	296	15 (1)	78 (63–87)	77 (62–86)
Relaxed vaccination history requirement^¶^						
No 2024–2025 COVID-19 dose (Ref)	**44,314**	422 (289–695)	1,575	1,312 (98)	Ref	Ref
≥ 1 2024–2025 COVID-19 dose, 7–179 days earlier	**3,029**	73 (38–112)	179	32 (2)	65 (50–75)	66 (51–76)

**Abbreviations:** Ref = referent group; VE = vaccine effectiveness.

* VE was calculated as (1 − adjusted odds ratio) × 100%, estimated using a test-negative case-control design, and adjusted for age in years, race and ethnicity, sex, calendar day (days since August 29, 2024), and geographic region with age and calendar day included as natural splines.

^†^ In the primary VE and extended VE analyses, the vaccinated group was children who completed an initial series with ≥1 2024–2025 dose as part of the series or who completed an initial series and then received a 2024–2025 dose as an additional vaccine. The comparator group was children who completed an initial COVID-19 vaccine series with no receipt of a 2024–2025 dose or with no recorded COVID-19 vaccinations. Children aged 9 months–4 years with an incomplete initial series were excluded from the primary analysis. Among vaccinated children aged 9 months–4 years, the initial series and 2024–2025 dose were not required to be from the same manufacturer.

^§^ The extended VE analysis included 44,889 total encounters, of which 1,295 (3%) involved case-patients.

^¶^ In this sensitivity VE analysis, the vaccinated group was children who received at least 1 2024–2025 dose, regardless of COVID-19 vaccination history; the comparator group was children with no receipt of a 2024–2025 dose, regardless of COVID-19 vaccination history. This analysis included 47,343 total encounters, of which 1,344 (3%) involved case-patients.

### 2024–2025 COVID-19 VE Against COVID-19–Associated ED/UC Visits in Children and Adolescents Aged 5–17 Years

Among children and adolescents aged 5–17 years, 53,467 ED/UC encounters met criteria for inclusion in the analyses, including 1,325 (2%) case-patients and 52,142 (98%) control patients (Table 1). Twenty-six (2%) case-patients and 2,462 (5%) control patients had received a 2024–2025 COVID-19 vaccine dose. Effectiveness of a 2024–2025 COVID-19 vaccination against a COVID-19–associated ED/UC visit was 56% (95% CI = 35%–70%) during the first 7–179 days after vaccination, and 45% (95% CI = 25%–59%) during the first 7–299 days after vaccination (Table 3). Results were similar when stratified by age (51% among children aged 5–11 years and 61% among children and adolescents aged 12–17 years, with overlapping CIs).

**TABLE 3:** Vaccine effectiveness* against laboratory-confirmed COVID-19–associated emergency department or urgent care encounters among children and adolescents aged 5–17 years — VISION, August 29, 2024–September 2, 2025

COVID-19 vaccination pattern	Total encounters, no.	Days since last dose among vaccinated persons, no.		Positive SARS-CoV-2 results, no. (column %)	VE, % (95% CI)	
		Median (IQR)	Maximum		**Unadjusted**	Adjusted*
**Primary VE estimates**						
No 2024–2025 COVID-19 dose (Ref)	**50,979**	986 (722–1,135)	1,633	1,299 (98)	Ref	Ref
At least 1 2024–2025 COVID-19 dose, 7–179 days earlier	**2,488**	84 (44–124)	179	26 (2)	60 (40–73)	56 (35–70)
**Sensitivity VE estimates^†^**						
Extended VE interval						
No 2024–2025 COVID-19 dose (Ref)	**50,979**	986 (722–1,135)	1,633	1,299 (97)	Ref	Ref
At least 1 2024–2025 COVID-19 dose, 7–299 days earlier	**3,152**	105 (55–167)	299	44 (3)	46 (27–60)	45 (25–59)
**Estimates by age group, yrs**						
5–11						
No 2024–2025 COVID-19 dose (Ref)	**31,508**	883 (595–1,050)	1,633	645 (98)	Ref	Ref
At least 1 2024–2025 COVID-19 dose, 7–179 days earlier	**1,443**	85 (44–126)	179	13 (2)	57 (24–75)	51 (14–72)
12–17						
No 2024–2025 COVID-19 dose (Ref)	**19,471**	1,056 (835–1,191)	1,552	654 (98)	Ref	Ref
At least 1 2024–2025 COVID-19 dose, 7–179 days earlier	**1,045**	82 (44–122)	179	13 (2)	64 (37–79)	61 (31–78)

**Abbreviations:** Ref = referent group; VE = vaccine effectiveness.

* VE was calculated as (1 – adjusted odds ratio) × 100%, estimated using a test-negative case-control design, and age in years, race and ethnicity, sex, calendar day (days since August 29, 2024), and geographic region with age and calendar day included as natural splines. In each analysis, the vaccinated group was children who received at least 1 2024–2025 COVID-19 vaccine dose, regardless of previous COVID-19 vaccination history; the comparator group was children with no receipt of a 2024–2025 COVID-19 vaccine dose, regardless of previous COVID-19 vaccination history.

^†^ The extended VE analysis included 54,131 total encounters, of which 1,343 (2%) involved case-patients.

## Discussion

The 2024–2025 COVID-19 vaccines provided protection against COVID-19–associated ED/UC encounters among children and adolescents aged 9 months–17 years. This evaluation included children and adolescents with varied COVID-19 vaccination and SARS-CoV-2 infection histories, and therefore, results should be interpreted as estimates of the additional protection provided by a 2024–2025 COVID-19 vaccine in a population with mixed preexisting immunity.

Infants aged 6–11 months have the highest rates of COVID-19–associated hospitalization of any COVID-19 vaccine–eligible pediatric age group, and COVID-19–associated hospitalization rates in the United States during the 2024–25 respiratory virus season were higher in this group than all adult age groups other than those aged ≥65 years (*8*), underscoring potential benefits of COVID-19 vaccination in eligible infants. In this analysis, VE was highest in children aged 9 months–4 years, although CIs overlapped with older age groups. The apparent higher VE in younger children might be due to lower rates of previous SARS-CoV-2 infection.**** The primary estimates for VE in this analysis were similar to or higher than 2024–2025 VE estimates for adults in the United States (*9*); estimates were also similar to or higher than those for 2023–2024 in children (35% [95% CI = 16%–49%] for children aged 9 months–4 years and 44% [95% CI = 29%–55%] for children and adolescents aged 5–17 years) (*6*). Higher estimates for the 2024–25 season might be due to different patterns of recent previous SARS-CoV-2 infection compared with the 2023–24 season or might be due to fewer changes in circulating SARS-CoV-2 variants during the 2024–25 season.

Vaccination based on shared clinical decision-making is individually based and guided by a decision process between the health care provider and the patient or parent/guardian; generally, ACIP recommendations adopted by CDC and listed on CDC immunization schedules, including those based on shared clinical decision-making, are covered by health insurance plans. The impact that shifting from universal to shared clinical decision-making (otherwise known as individual-based decision-making) will have on COVID-19 vaccination coverage or effectiveness in children is unclear, underscoring the importance of continued monitoring of COVID-19 VE.

### Limitations

The findings in this report are subject to at least five limitations. First, although case-patients met a COVID-19–like illness definition and received a positive SARS-CoV-2 test result, they might have visited ED/UCs for reasons other than COVID-19, potentially lowering VE estimates. Second, misclassification of vaccination status was possible, which would likely result in underestimation of VE if the misclassification was nondifferential. Previous estimates across networks including various COVID-19 vaccine history ascertainment methods (i.e., EHR, immunization information systems, self-report, and claims data) have yielded similar VE estimates (*9*). Third, children aged 9 months–4 years and children and adolescents aged 5–17 years account for a smaller fraction of the general population than adults in age groups frequently examined in VE analyses (i.e., 18–64 years and ≥65 years), decreasing the sample size available for estimating VE in children and adolescents compared with adults. In addition, because of relatively low COVID-19 vaccination coverage in children compared with adults and overall lower rates of medically attended COVID-19 during 2024–2025 compared with 2023–2024, this study did not have sufficient statistical power to measure VE by finer intervals of time since dose and for hospitalization. Fourth, although analyses were adjusted for some relevant confounders, residual confounding from other factors, such as behavioral modifications to prevent SARS-CoV-2 exposure and outpatient antiviral treatment for COVID-19, might remain. Finally, low COVID-19 vaccination coverage among children and adolescents might reduce the generalizability of results.

### Implications for Public Health Practice

In this analysis, receipt of a 2024–2025 COVID-19 vaccine dose provided additional protection against COVID-19–associated ED/UC visits among children and adolescents aged 9 months–17 years in a population with preexisting levels of protection from previous vaccination, previous infection, or both. CDC continues to monitor VE of COVID-19 vaccines.

## Footnotes

* Variants and Genomic Surveillance | COVID-19 | CDC (Accessed November 14, 2025).

†
FDA Approves and Authorizes Updated mRNA COVID-19 Vaccines to Better Protect Against Currently Circulating Variants | FDA

§ The recommendation for unvaccinated children and adolescents aged ≥12 years receiving the Novavax COVID-19 vaccine is 2 doses.

¶
ACIP Shared Clinical Decision-Making Recommendations | ACIP | CDC

**
Child Immunization Schedule Notes | Vaccines & Immunizations | CDC

†† Minimum age of 6 months at first dose and a minimum of 11 weeks between doses 1 and 3.

§§ Eligible ED/UC encounters or hospital admissions were those for COVID-19–like illness, obtained using *International Classification of Diseases, Tenth Revision* (ICD-10) discharge codes. The specific codes used were COVID-19 pneumonia: J12.81 and J12.82; influenza pneumonia: J09.X1, J10.0*, J11.0*, and other viral pneumonia: J12*; bacterial and other pneumonia: J13, J14, J15*, J16*, J17, and J18*; influenza disease: J09*, J10.1, J10.2, J10.8*, J11.1, J11.2, and J11.8*; acute respiratory distress syndrome: J80; chronic obstructive pulmonary disease with acute exacerbation: J44.1; acute asthma exacerbation: J45.21, J45.22, J45.31, J45.32, J45.41, J45.42, J45.51, J45.52, J45.901, and J45.902; respiratory failure: J96.0*, J96.2*, R09.2, and J96.9*; other acute lower respiratory tract infections: B97.4, J20*, J21*, J22, J40, J44.0, J41*, J42, J43*, J47*, J85*, and J86*; acute and chronic sinusitis: J01* and J32*; acute upper respiratory tract infections: J00*, J02*, J03*, J04*, J05*, and J06*; acute respiratory illness signs and symptoms: R04.2, R05, R05.1, R05.2, R05.4, R05.8, R05.9, R06.00, R06.02, R06.03, R06.1, R06.2, R06.8, R06.81, R06.82, R06.89, R07.1, R09.0*, R09.1, R09.2, R09.3, and R09.8*; acute febrile illness signs and symptoms: R50*, R50.81, R50.9, and R68.83; acute nonrespiratory illness signs and symptoms: M79.10, M79.18, R10.0, R10.1*, R10.2, R10.3*, R10.81*, R10.84, R10.9, R11.0, R11.10, R11.11, R11.15, R11.2, R19.7, R21*, R40.0, R40.1, R41.82, R43*, R51.9, R53.1, R53.81, R53.83, R57.9, and R65*; febrile convulsions: R56.0; viral and respiratory diseases complicating pregnancy, childbirth, and puerperium: O98.5*, O98.8*, O98.9*, and O99.5*. All ICD-10 codes with * include all child codes under the specific parent code.

¶¶ National pharmacy chains were required to establish bidirectional linkage with jurisdictional immunization information systems (IISs) to support vaccine distribution early in the COVID-19 pandemic; thus, doses administered at pharmacies should be reported to IISs.

*** Antigen tests generally have lower sensitivity and specificity than molecular tests. In the test-negative design, small reductions in test specificity can cause greater bias in VE estimates than more substantial reductions in test sensitivity.

††† Earlier of ED/UC encounter or respective SARS-CoV-2 testing date.

§§§ Geographic region was included in the model based on site of the final discharge facility of the encounter.

¶¶¶ 45 C.F.R. part 46.102(l)(2), 21 C.F.R. part 56; 42 U.S.C. Sect. 241(d); 5 U.S.C. Sect. 552a; 44 U.S.C. Sect. 3501 et seq.

****
COVID-19 Vaccine Effectiveness Update | CDC

## References

[1] Wiegand RE, Devine O, Wallace M, Estimating COVID-19 associated hospitalizations, ICU admissions, and in-hospital deaths averted in the United States by 2023-2024 COVID-19 vaccination: a conditional probability, causal inference, and multiplier-based approach. Vaccine 2025;49:126808. 10.1016/j.vaccine.2025.126808 39889531 PMC12168029

[2] Panagiotakopoulos L, Moulia DL, Godfrey M, Use of COVID-19 vaccines for persons aged ≥6 months: recommendations of the Advisory Committee on Immunization Practices—United States, 2024–2025. MMWR Morb Mortal Wkly Rep 2024;73:819–24. 10.15585/mmwr.mm7337e2 39298394 PMC11412444

[3] Thompson MG, Stenehjem E, Grannis S, Effectiveness of Covid-19 vaccines in ambulatory and inpatient care settings. N Engl J Med 2021;385:1355–71. 10.1056/NEJMoa2110362 34496194 PMC8451184

[4] Klein NP, Demarco M, Fleming-Dutra KE, Effectiveness of BNT162b2 COVID-19 vaccination in children and adolescents. Pediatrics 2023;151:e2022060894. 10.1542/peds.2022-060894 37026401

[5] Rowley EAK, Mitchell PK, Yang D-H, Methods to adjust for confounding in test-negative design COVID-19 effectiveness studies: simulation study. JMIR Form Res 2025;9:e58981. 10.2196/58981 39869907 PMC11811671

[6] Irving SA, Rowley EAK, Chickery S, Effectiveness of 2024–2025 COVID-19 vaccines in children in the United States. Pediatrics 2025;156:e2025073212. 10.1542/peds.2025-073212 41224092 PMC13297095

[7] Payne AB, Ciesla AA, Rowley EAK, ; VISION Network. Impact of accounting for correlation between COVID-19 and influenza vaccination in a COVID-19 vaccine effectiveness evaluation using a test-negative design. Vaccine 2023;41:7581–6. 10.1016/j.vaccine.2023.11.025 38000964 PMC11823735

[8] CDC. Updates to COVID-19 epidemiology [Presentation slides]. Presented at the Advisory Committee on Immunization Practices meeting, Atlanta, GA; September 19, 2025. https://www.cdc.gov/acip/downloads/slides-2025-09-18-19/02-Srinivasan-covid-508.pdf.

[9] Link-Gelles R, Chickery S, Webber A, ; CDC COVID-19 Vaccine Effectiveness Collaborators. Interim estimates of 2024–2025 COVID-19 vaccine effectiveness among adults aged ≥18 years—VISION and IVY networks, September 2024–January 2025. MMWR Morb Mortal Wkly Rep 2025;74:73–82. 10.15585/mmwr.mm7406a1 40014628 PMC11867580

